# A base-calling algorithm for Tm-shifted melting curve SNP assay

**DOI:** 10.1186/2043-9113-1-3

**Published:** 2011-01-20

**Authors:** Kung-Hao Liang, Jun-Jeng Fen, Hsien-Hsun Chang, Hsei-Wei Wang, Yuchi Hwang

**Affiliations:** 1 Vita Genomics Inc., Jungshing Road, Taipei County, 248 Taiwan; 2 Institute of Biomedical Informatics, National Yang-Ming University, Linong Street, Taipei, 112 Taiwan; 3 Graduate Institute of Biomedical Materials and Engineering, Taipei Medical University, Wu-Hsing Street, Taipei, 110 Taiwan; 4 Institute of Microbiology and Immunology, National Yang-Ming University, Li-Nong Street, Taipei, 112 Taiwan

## Abstract

**Background:**

Tm-shifted melting curve SNP assays are a class of homogeneous, low-cost genotyping assays. Alleles manifest themselves as signal peaks in the neighbourhood of theoretical allele-specific melting temperatures. Base calling for these assays has mostly relied on unsupervised algorithm or human visual inspection to date. However, a practical clinical test needs to handle one or few individual samples at a time. This could pose a challenge for unsupervised algorithms which usually require a large number of samples to define alleles-representing signal clusters on the fly.

**Methods:**

We presented a supervised base-calling algorithm and software for Tm-shifted melting curve SNP assays. The algorithm comprises a peak detection procedure and an ordinal regression model. The peak detection procedure is required for building models as well as handling new samples. Ordinal regression is proposed because signal intensities of alleles AA, AB, and BB usually follow an ordinal pattern with the heterozygous allele lie between two distinct homozygous alleles. Coefficients of the ordinal regression model are first trained and then used for base calling.

**Results:**

A dataset of 12 SNPs of 44 unrelated persons was used for a demonstration purpose. The call rate is 99.6%. Among the base calls, 99.1% are identical to those made by the sequencing method. A small fraction of the melting curve signals (0.4%) is declared as "no call" for further human inspection. A software was implemented using the Java language, providing a graphical user interface for the visualization and handling of multiple melting curve signals.

**Conclusions:**

Tm-shifted melting curve SNP assays, together with the proposed base calling algorithm and software, provide a practical solution for genetic tests on a clinical setting. The software is available in http://www.bioinformatics.org/mcsnp/wiki/Main/HomePage

## Background

Discoveries of associations between genetic variants and clinical traits have improved our knowledge of human in health and disease [1]. Most of these findings came from research-phrase genome-wide association studies (GWAS) of various common-complex diseases [2-5]. Once validated in independent cohorts, these associations can facilitate the development of genetic tests for estimating personal disease risks. As GWAS gains popularity among clinical scientists, genetic tests are anticipated to play an increasingly important role in preventive and personalized healthcare systems.

Single nucleotide polymorphism (SNP) is an important class of human genomic variants widely assayed on GWAS. Current genetic tests are constructed on high-density genome-wide assays [6] or low-cost, SNP-specific assays. The former aims to provide an extensive list of disease reports, while the latter gives results pertaining to a particular disease or a clinical trait.

A variety of assays has been developed for genotyping SNPs on the human DNA [7,8]. For research-phase projects, samples are usually collected in panels of many reaction wells and analyzed using unsupervised base calling algorithms. The entire panel is usually designated for a particular SNP. The fluorescent intensity signal of the entire panel is then clustered on-the-fly to make calls (e.g. [5] and the Rotor-Gene ScreenClust HRM Software). All three alleles of the SNP need to exist in the panel to define clusters properly. For cases when one allele type is rare, a larger pool of samples may be required to make the rare allele well represented [8]. In practice, many clinical labs received samples individually, each requiring the results to be delivered as soon as possible. Consequently, it is more practical and cheaper to run different assays (for different SNPs and/or different persons) concurrently in the same panel. Different SNPs may have different SNP-specific fluorescent distributions, prohibiting themselves to be clustered together. Therefore, a supervised base calling algorithm may be more adequate in a clinical setting. The SNP-specific coefficients are pre-trained to facilitate the base calling of individual samples.

The melting curve SNP genotyping assay, abbreviated as McSNP, is a class of simple, fast and relatively low-cost assays [9-19]. Among them, the Tm-shifted methods employ allele-specific primers which are designed to increase the melting temperature (Tm) difference between two allele-specific PCR duplex [14,18,19]. They are homogeneous assays where the entire process, including amplification and detection, is performed in solution within a single reaction well. Each allele manifested differently at its particular Tm. The base calling of Tm-shifted McSNP technology has relied mostly on unsupervised algorithm [18], user-specified cut-offs [16] or human visual inspection to date. Hence, we were motivated to propose a supervised base calling algorithm, enabling the McSNP assay a practical genetic test.

Denote the two alleles of a haploid SNP as A and B respectively. The goal of a base calling algorithm is to identify whether the assayed diploid SNP is homozygous AA (allele 1), heterozygous AB (allele 2), or homozygous BB (allele 3). Signals of AA, AB and BB usually follow a sequential order on a variety of assays including McSNP. Hence, we proposed an algorithm which comprises two procedures: (1) peak detection; and (2) base calling by an ordinal regression model. The peak detection procedure is required for both model training and the actual base calling. We also proposed the use SNP-specific offsets for adequate adjustments of the model to accommodate SNP-specific signal strengths. Samples of known alleles (determined by the conventional sequencing method) were used to train the coefficients of the algorithm, including the SNP-specific offsets and the ordinal regression coefficients. The trained model can then used for handling new coming samples.

## Methods

### The Tm-shifted McSNP assay

There are several variants of Tm-shifted McSNP assay [14,18,19]. We followed the protocol in [14] for primer design and experiment setting as an example. This technique requires two forward primers and one common reverse primer. The three primers form two primer pairs, amplifying allele-specific PCR products containing alleles A and B respectively. Reagents comprised SYBR Green PCR Master Mix (Applied Biosystem #4309155) (6 μL), two forward and one reverse SNP-specific primers (0.4 μM each), and the human genomic DNA (20 ng). The total reaction volume was 10 μL.

The assay started with a PCR procedure for DNA amplification. This started form the pre-incubation at 95°C to activate the Taq DNA polymerase (10 mins), followed by 50 cycles of thermal cycling comprising (1) denaturation at 95°C (15s) and (2) primer annealing and extension at 60°C (1 min). Afterwards, we continued the dissociation of the DNA duplex by gradually increasing the temperature up to 95°C at a temperature gradient of 0.2°C/min.

The Applied Biosystems ABI 7900HT instrument was used. The fluorescent signal was captured by the accompanied SDS 2.2 software. The theoretical temperature Tm was calculated using the dnaMate server [20] where a consensus melting temperature was calculated using the nearest-neighbour model based on three independent thermodynamic tables.

### Signal processing and peak Detection

A disassociation curve, denoted as *F(T)*, is the fluorescent intensity plot captured during a dissociation process with increasing temperature *T *. Define a melting curve *M *as the negative first-derivative of the disassociation curves *F *[13], therefore

M=–dF/dT

**Figure 1:**
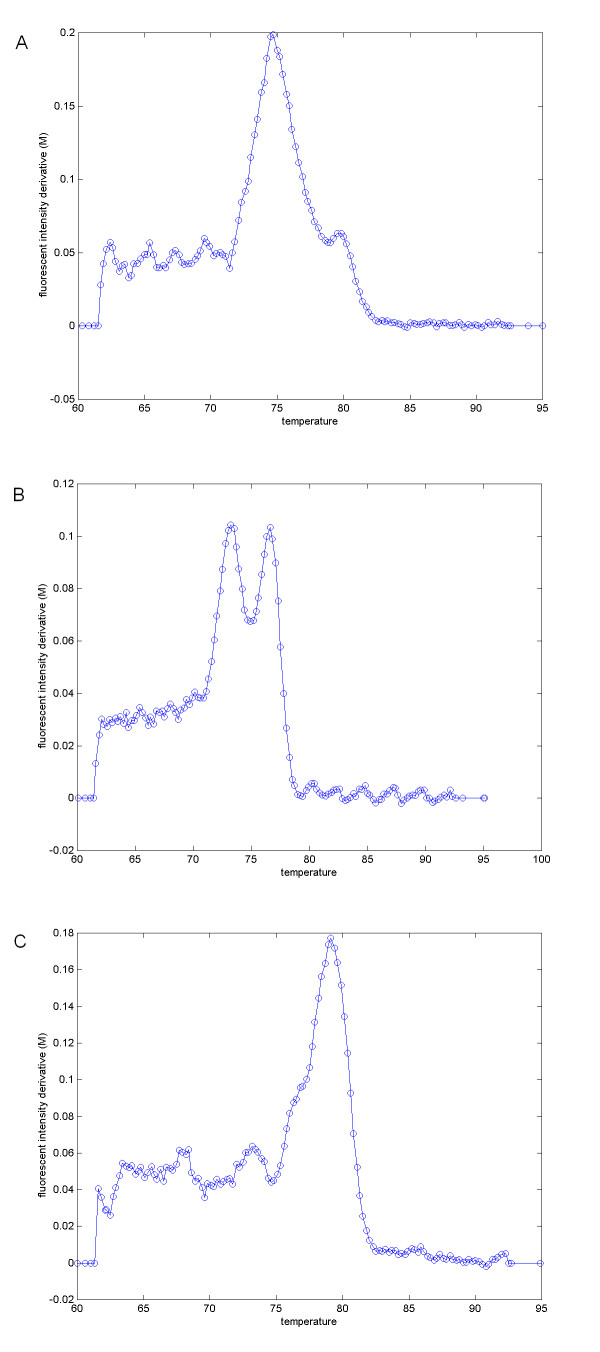
**Typical melting curve plots of three alleles**. (A) allele 1; (B) allele 2; (C) allele 3. The horizontal axis represents the temperature (*T*). The vertical axis is the fluorescent intensity derivative (*M*) w.r.t. temperature. The major peaks of the curve occur in the proximity of theoretical melting temperatures of the two allele-specific PCR duplex.

Denote *Tm(A) *and *Tm(B) *as the theoretical melting temperatures of the PCR products, where *Tm(A) *<*Tm(B)*. Alleles manifest themselves as peaks on *M *occurring near *Tm(A) *and *Tm(B)*. Figure 1 illustrates the typical melting curve signals of the three types of alleles. A single peak on *M *indicates a homozygous allele (Figure 1a and 1c), while two peaks indicate a heterozygous allele (Figure 1b). An optional Gaussian smoothing is applied to *M *to suppress the small noisy fluctuations of the signal while preserving the major bending curves on *M*.

The proximity of *Tm(A) *and *Tm(B)*, denoted as *R(A) *and *R(B) *respectively, are the main target regions of peak searching. This allows some degree of variation of the real *Tm *from the theoretical *Tm*.

$$\begin{array}{l} R_{A}=\left(2*Tm\left(A\right)-Tm\left(B\right),\left(Tm\left(A\right)+Tm\left(B\right)\right)/2\right]\\ R_{B}=\left(\left(Tm\left(A\right)+Tm\left(B\right)\right)/2,2*Tm\left(B\right)-Tm\left(A\right)\right] \end{array}$$

A derivative of the melting curve is then calculated within *R*_*A *_and *R*_*B*_. A zero-crossing of the derivative either represents a peak (convex) or a valley (concave) on the melting curve. The peaks and valleys of a region are compared based on their height to find the tallest peak. The signal strengths of *A *and *B *alleles, denoted as *D*_*A *_and *D*_*B *_respectively, are the heights of the tallest peaks on *R*_*A *_and *R*_*B*_, deducting the average height of the entire curve for normalization purposes. *D*_*A *_or *D*_*B *_takes the value of zero if no peak is detected in the corresponding region. If both *D*_*A *_and *D*_*B *_are 0, then a "no call" is reported. Otherwise, a variable *x *is introduced as the ratio of signal strengths:

$$x=D_{B}/(D_{A}+D_{B})$$

### The ordinal regression model for base calling

The base calling model was built upon the ordinal regression method, taking advantage of the fact that signal patterns of AA, AB and BB usually follow a sequential order, with the heterozygous allele lie between two distinct homozygous alleles. Alleles 1 (AA), 2 (AB) and 3 (BB) constitute the three ordered categories of the response variable *Z *of the regression model. Our implementation has three model coefficients*α1*, *α2 *and *β*. Given the coefficients, the cumulative response probabilities when *Z ={allele 1} *(denoted as *P(Z ={1})) *and *Z ={alleles 1,2} *(denoted as *P(Z ={1,2}))*, can be estimated using the following equations.

$$\begin{array}{l} logit\left(P\left(Z=\{1\}\right)\right)=\alpha 1-\beta X\\ logit\left(P\left(Z=\{1,2\}\right)\right)=\alpha 2-\beta X \end{array}$$

The individual allele probability functions of alleles 2 and 3 can then be calculated by

$$\begin{array}{c} P\left(Z=\{2\}\right)=P\left(Z=\{1,2\}\right)-P\left(Z=\{1\}\right)\\ P\left(Z=\{3\}\right)=1-P\left(1,2\right) \end{array}$$

A probability margin ρwas introduced. Bases are called by the following rules:

   If ((P(*Z={2}*)-P(*Z={1}*))>ρ & (P(*Z={2}*)-P(*Z={3}*))>ρ)

      "Allele 2";

   else if ((P(*Z={3}*)-P(*Z={1}*))>ρ & (P(*Z={3}*)-P(*Z={2}*))>ρ)

      "Allele 3";

   else if ((P(*Z={1}*)-P(*Z={2}*))>ρ & (P(*Z={1}*)-P(*Z={3}*))>ρ)

      "Allele 1";

   else "no call"

If the difference the top two probabilities is smaller than ρ, then the base is called "no call" so as to trigger a warming message for human inspection.

## Results and Discussion

### Determining coefficients

The algorithm was trained on 44 human samples for a demonstration of this algorithm. Samples were from healthy Asian volunteers who has sign the inform consent form. Each sample was genotyped on a set of 12 SNPs (Table 1), producing 528 melting curve plots in total. The signal strength ratio *x *was calculated for each plot (see **Methods**). These samples were also genotyped by the conventional sequencing method, serving as the expected calling results.

**Table 1:** List of SNPs

ID	Gene Symbol	SNP	Allele (A/B)
SNP1		rs2241796	T/C
SNP2	TGFBRAP1	rs1866040	G/A
SNP3		rs2576737	A/G
SNP4		rs518604	C/T
SNP5	CASP5	rs2282658	C/G
SNP6		rs484345	A/G
SNP7		rs1699087	G/T
SNP8	ADAR	rs903323	T/C
SNP9	IFI44	rs2070123	T/C
SNP10		rs305067	G/C
SNP11	ICSBP1	rs305088	A/G
SNP12		rs870614	G/A

These SNPs were assayed by both the sequencing and the McSNP methods for the demonstration of proposed algorithm.

**Figure 2:**
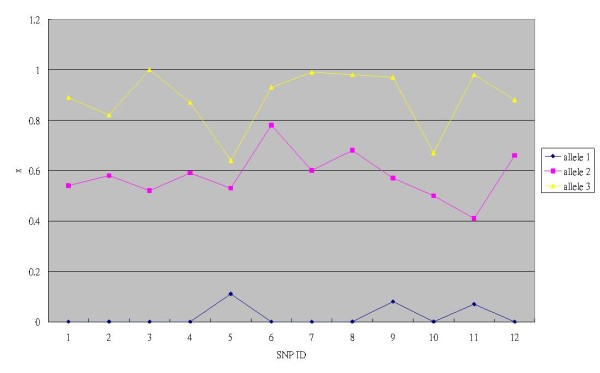
**Allele-specific signal strength ratio (*x*) derived from melting curves**. Average *x *of alleles 1, 2 and 3 for each of the 12 SNPs.

We aimed to obtain general coefficients rather than SNP specific coefficients to suit multiple SNPs. However, variations of *x *do occur between different SNPs. Figure 2 shows the averages of *x *for each allele of the 12 SNPs. To accommodate the variations of *x*, a SNP-specific offset δ is introduced which is calculated as follows. First, we take grand means *〈x〉 *of the SNP-specific averages across all the 12 SNPs for alleles 1, 2 and 3. Second, δ's are calculated by the SNP-specific averages of *x *minus the grand means *〈x〉*. We hoped to maintain zero offsets for most SNPs, therefore, the offsets were purposely kept in low resolution. They were rounding off to one decimal digit. As a consequence, 8 SNPs have zero offsets; SNPs 6 and 8 have an offset of 0.1. SNPs 5 and 10 have an offset of -0.1.

**Figure 3:**
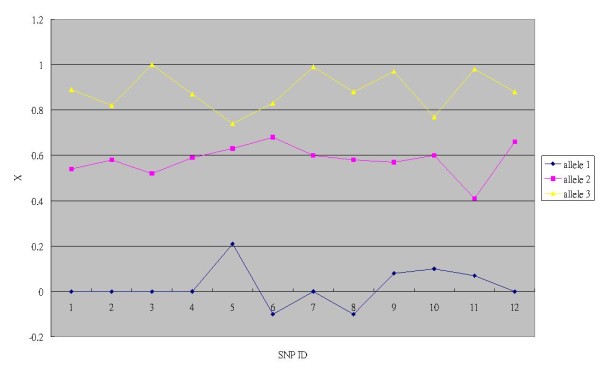
**Adjusted signal strength ratio (*X*)**. Average *X *of alleles 1, 2 and 3 for each of the 12 SNPs. SNPs 5, 6, 8 and 10 are offset from *x *in Figure 2.

**Figure 4:**
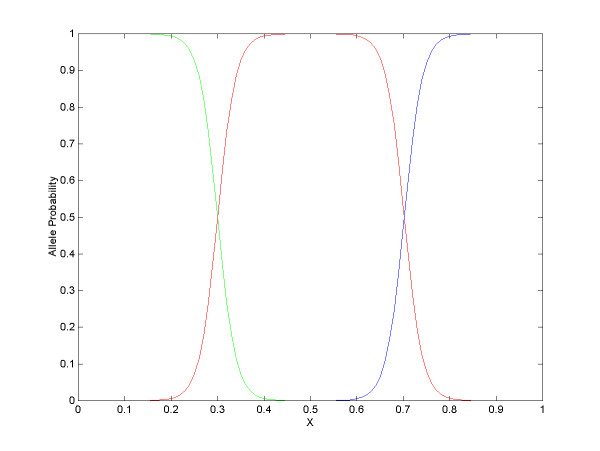
**Allele probability functions**. Allele probability, a function of *X*, is given by the ordinal regression model. Green: allele 1. Red: allele 2. Blue: allele 3.

We further introduced the adjusted signal strength ratio *X*, defined as *X = x -*δ. Compared with *x*, the distributions of *X *of the 12 SNPs resemble each other better (Figure 3). Hence, *X *is used for building the ordinal regression model. Based on all the 528 plots, *α1 *= 15.3, *α2 *= 35.8, *b *= 51. The resulting allele probability functions *P(Z = {1}), P(Z = {2}) *and *P(Z = {3}) *are shown in Figure 4 which is the basis for subsequent base calling.

X and x is only different by an offset*δ *which takes one of three values, -0.1, 0 and 0.1. Referring to the ordinal regression equation:

$$\begin{array}{c} logit\left(P\left(Z=\{1\}\right)\right)=\alpha 1-\beta X\\ =\alpha 1-\beta (x-\delta )\\ =(\alpha 1-\delta )\;–\beta x, \end{array}$$

the three offsets effectively generates three different models to accommodate the variation of signal strength ratios of the 12 SNPs. The model with zero offsets may have the widest use because it is built upon a large portion of the training dataset.

### Base calling performance

The margin of probability ρ was set at 0.05 for the base calling. The performance was summarized in Table 2. The call rate is 99.6% because two SNPs are declared as no calls. Among the 12 SNPs, 10 SNPs reached 100% concordance rate, defined as the percentage of base calls identical to those from the sequencing method. The average concordance rate is 99.1%. For all the discordant callings, base calls by the sequencing method were allele 2, while by McSNP were allele 3 (Table 3). This is because the melting-curve signals on the first allele is relatively weak, occasionally missing, thus the first alleles are not easily detected by the base calling algorithm.

**Table 2:** SNP-specific calling performance

	SNP 1	SNP 2	SNP 3	SNP 4	SNP 5	SNP 6	SNP 7	SNP 8	SNP 9	SNP 10	SNP 11	SNP 12
No calls	0	0	0	0	0	1	1	0	0	0	0	0

# discordant calls	0	0	0	0	1	4	0	0	0	0	0	0

Concordance rate (%)	100	100	100	100	97.7	90.7	100	100	100	100	100	100

The number of no calls, discordant calls and the concordance rates between the proposed algorithm and the sequencing method

**Table 3:** Comparison of the discordant calls between McSNP and sequencing.

	McSNP	Sequencing	*X*	P(allele 1)	P(allele 2)	P(allele 3)
SNP5	allele 3 (CC)	allele 2 (CG)	0.73	0	0.19	0.81
SNP6	allele 3 (AA)	allele 2 (AG)	0.71	0	0.40	0.60
SNP6	allele 3 (AA)	allele 2 (AG)	0.72	0	0.28	0.72
SNP6	allele 3 (AA)	allele 2 (AG)	0.71	0	0.40	0.60
SNP6	No call	allele 2 (AG)	0.70	0	0.52	0.48
SNP6	allele 3 (AA)	allele 2 (AG)	0.71	0	0.40	0.60
SNP7	No call	allele 2 (GT)	0.70	0	0.52	0.48

Base calls, allele signals (X) and their corresponding allele probabilities are presented.

### The software

**Figure 5:**
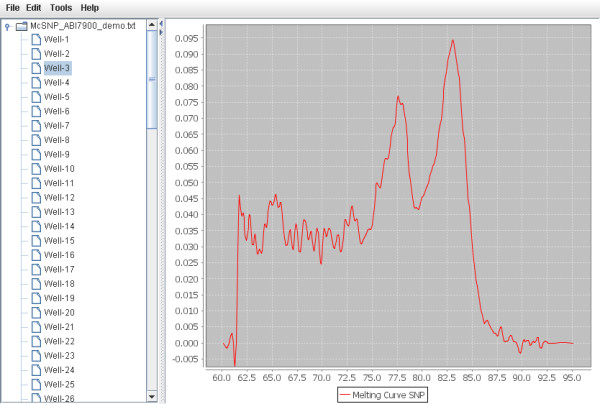
**The graphical user interface of the software**. The software was implemented in Java for providing a convenient interface for data visualization and handling.

A software was developed on the Java programming language to implement the proposed algorithm and also provide a user friendly graphical interface. The software can handle a fluorescent signal exports from SDS2.2 and then calculate the signal strength ratio *x*. Given SNP-specific offsets, theoretical melting temperatures and the coefficients of the ordinal regression model, the software can then make calls. The graphical user interface was designed for the ease of signal visualization and manipulation (Figure 5). The software is available in http://www.bioinformatics.org/mcsnp/wiki/Main/HomePage.

## Conclusions

The supervised base calling algorithm and software were designed for the clinical use of Tm-shifted melting curve SNP genotyping assays. A supervised algorithm was designed due to practical considerations of its clinical use. An ordinal regression model was employed to capture the sequential order of average allele signals. A set of general coefficients were provided based on a demonstration dataset. Clinicians can conduct the base calling using the general coefficients, or carry out the coefficients training and the subsequent base calling themselves

Although this algorithm was developed upon the Tm-shifted McSNP data, it can be adapted for other McSNP methods. Particularly, this line of technology is still evolving and new improvements of the analytical chemistry appear gradually. The proposed algorithm and training strategy can also evolve accordingly. By the combination of efficient base calling software and a small-scale human inspection, a practical SNP tests can be established.

## Competing interests

The authors declare that they have no competing interests.

## Authors' contributions

KHL designed the algorithm, implemented the prototype of the core algorithm and drafted the manuscript. JJF implemented the JAVA version of the software with friendly graphical user interface. HHC conducted the McSNP and sequencing experiments. HWW contributed on the study design and data analysis. YCH conceived and coordinated the study. All authors read and approved the final manuscript.
